# Zika Virus and Guillain–Barre Syndrome: Is There Sufficient Evidence for Causality?

**DOI:** 10.3389/fneur.2016.00170

**Published:** 2016-09-30

**Authors:** A. Arturo Leis, Dobrivoje S. Stokic

**Affiliations:** ^1^Center for Neurosciences and Neurological Recovery, Methodist Rehabilitation Center, Jackson, MS, USA; ^2^Department of Neurology, Mayo Clinic, Scottsdale, AZ, USA

**Keywords:** Zika virus, Guillain–Barre syndrome, acute flaccid paralysis, neurotropic virus, electrodiagnostic studies, nerve conduction studies, electromyography

## Abstract

Worldwide concern over Zika virus causing Guillain–Barre syndrome (GBS) soared after recent reports that Zika-related weakness was due to GBS. A global strategic response plan was initiated with recommendations for at-risk countries to prepare for GBS. This plan has major economic implications, as nations with limited resources struggle to implement costly immunotherapy. Since confirmation of causality is prerequisite to providing specific management recommendations, it is prudent to review data endorsing a GBS diagnosis. We searched PubMed for manuscripts reporting original clinical, laboratory, and electrodiagnostic data on Zika virus and GBS. Five papers met criteria; four case reports and one large case–control study (French Polynesia) that attributed 42 paralysis cases to a motor variant of GBS. Brighton criteria were reportedly used to diagnose GBS, but no differential diagnosis was presented, which violates criteria. GBS was characterized by early onset (median 6 days post-viral syndrome), rapid progression (median 6 days from onset to nadir), and atypical clinical features (52% lacked areflexia, 48% of facial palsies were unilateral). Electrodiagnostic evaluations fell short of guidelines endorsed by American Academy of Neurology. Typical anti-ganglioside antibodies in GBS motor variants were rarely present. We conclude that there is no causal relationship between Zika virus and GBS because data failed to confirm GBS and exclude other causes of paralysis. Focus should be redirected at differential diagnosis, proper use of diagnostic criteria, and electrodiagnosis that follows recommended guidelines. We also call for a moratorium on recommendations for at-risk countries to prepare costly immunotherapies directed at GBS.

## Introduction

Zika virus is a neurotropic Flavivirus that can cause disease within the central nervous system (CNS). The neurotropism of Flaviviruses is well-documented, and cases of acute flaccid paralysis due to myelitis with or without brainstem involvement have been reported with Dengue (1), Japanese encephalitis (2), Central European encephalitis (3), West Nile virus (4–6), and most recently, Zika in French West Indies (7) and French Polynesia (8). Moreover, in some newborns with microcephaly, brain tissue is positive for Zika virus RNA by reverse transcription-polymerase chain reaction (RT-PCR) assays, confirming the strong neurotropism of this virus (9). In contrast, the peripheral nervous system is usually spared in Flavivirus infections. Notably, non-poliovirus myelitis caused by a neurotropic virus can mimic Guillain–Barre syndrome (GBS) (10, 11), and patients presenting with acute flaccid paralysis due to viral myelitis are frequently misdiagnosed with GBS or a motor variant of GBS, as occurred after West Nile virus gained entry into North America in 1999 (4). The acute flaccid paralysis associated with West Nile virus was erroneously attributed to GBS until late 2002, when electrodiagnostic and pathologic findings confirmed a poliomyelitis syndrome rather than a peripheral nerve disorder to explain the vast majority of acute flaccid paralysis cases (5, 6). Considering the known neurotropism of Zika virus and other Flaviviruses, the ease with which viral myelitis can be confused with variants of GBS, and the fact that no confirmed outbreak of GBS has ever occurred with Flaviviruses, there is an urgent need to clarify whether the neuromuscular manifestations of Zika virus arise primarily from the peripheral or CNS. This will avoid history repeating itself, since the acute flaccid paralysis associated with Zika virus infection, like West Nile virus, has recently been attributed to GBS. Reports of Zika virus causing GBS were quickly endorsed by editorial teams, public health organizations, and the world press (12–14), and Zika virus was hastily added to the list of pathogens that precipitate GBS (14, 15). By April 21, 2016, less than 2 months after the first published study describing a GBS outbreak (12), the World Health Organization (16) concluded that “based on a growing body of research, there is scientific consensus that Zika virus is a cause of GBS.” A strategic response plan was initiated, and at-risk countries were advised to prepare for GBS. That same month, the WHO reported over 600 new cases of Zika virus-related GBS in Latin American and Caribbean countries where gold-standards for confirming acute Zika virus infection and GBS were largely unavailable. Thus, precedence was quickly established for new cases of Zika virus acute flaccid paralysis to become Zika virus GBS, based solely or primarily on clinical grounds (17). This acceptance has major global economic implications, as nations with limited resources struggle to implement costly immunotherapy directed at a growing number of unconfirmed GBS cases. Since a clear understanding of the pathogenesis of Zika acute flaccid paralysis is a prerequisite to providing specific therapeutic and management recommendations, it is imperative and timely to review the data endorsing a diagnosis of GBS.

## Methodology

To this end, we performed a PubMed search using terms “Zika virus” and “GBS.” We also accessed information from national and international public health websites, including the Centers for Disease Control and Prevention,^1^ European Center for Disease Prevention and Control,^2^ and the WHO.^3^ As of June 28, 2016, the search returned 90 hits, which were reviewed for full text articles that reported original clinical, laboratory, and electrodiagnostic data on Zika virus and GBS.

## Observations/Discussion

Five papers met criteria for providing original data (Table 1); four were case reports that described a total of five cases of classic demyelinating GBS in French Polynesia (18), Puerto Rico (19), Martinique (20), and Brazil (21). In contrast, one paper was a large case–control study of a Zika virus outbreak in French Polynesia that attributed 42 cases of acute flaccid paralysis to the acute motor axonal neuropathy variant of GBS (12). This was the first and only study providing original clinical and electrodiagnostic evidence for Zika virus infection causing GBS, despite earlier suspicion of an association with GBS during the 2013–2014 outbreak in French Polynesia (8, 22). Co-authors of the case–control study also published a second paper that provided additional information on presumably the same 42 GBS cases (23). The implication of the case–control study was that at-risk countries needed to prepare to manage patients with the motor variant of GBS. A commentary accompanying this paper endorsed the diagnosis of GBS by concluding “Zika virus can be added to our list of viruses that can cause GBS,” but also stressed the need for a better understanding of the pathogenesis of this disease (15). The GBS outbreak in French Polynesia combined with new clusters of paralysis attributed to GBS in the Americas created a substantial spike in new cases of GBS that, in conjunction with microcephaly cases, prompted the WHO to declare a Public Health Emergency of International Concern in February 2016. Prior to the case–control study in French Polynesia, there were only two single case reports of the demyelinating form of GBS associated with Zika virus infection [(18, 19), Table 1]. These two preceding case reports, and the subsequent two case reports, had little impact on changing the global perspective on Zika virus and GBS. It is difficult to determine whether these case reports even reflected a genuine increase in GBS precipitated by Zika virus or the result of enhanced reporting. Hence, the large case–control study provided the only evidence supporting a GBS etiology for Zika-related acute flaccid paralysis. A recent update on Zika virus-associated neurological disorders, published 4 months after the WHO declared a Public Health Emergency, described the case–control study from French Polynesia (12) as “the only report with reliable information on the diagnostic criteria used to identify suspect GBS cases associated with Zika virus infection” (24). Accordingly, it is this study that warrants further scrutiny.

**Table 1:** **Five papers providing original clinical, laboratory, and electrodiagnostic data on Zika virus and Guillain–Barre syndrome**.

Reference	Publication date	Publication type	Number of cases	Geographic location	EDX: type of GBS	Zika virus confirmation	CSF results	Clinical manifestations
Oehler et al. (18)	March 6, 2014	Case report	1	French Polynesia	Demyelinating	(+) IgM, IgG; (+) PRNT; (−) RT-PCR serum	Cyto-albumin dissociation	Tetraparesis; areflexia; paresthesia; facial palsy (asymmetric); autonomic dysfunction
Thomas et al. (19)	February 12, 2016	Case report	1	Puerto Rico	Demyelinating	(+) IgM; (−) RT-PCR in serum and urine	Elevated protein (no details)	Tetraparesis; areflexia; facial palsy (bilateral); autonomic dysfunction
Cao-Lormeau et al. (12)	February 29, 2016	Case–control study	42	French Polynesia	AMAN	(+) IgM or IgG; (+) neutralizing antibodies; (−) RT-PCR in serum	Cyto-albumin dissociation	Tetraparesis or paraparesis; areflexia; paresthesia; facial palsy (33% bilateral, 31% unilateral); dysphagia
Rozé et al. (20)	March 3, 2016	Case report	2	Martinique	Demyelinating	(+) RT-PCR in urine	Cyto-albumin dissociation	Both cases: tetraparesis; areflexia; numbness; facial palsy (asymmetric); respiratory failure
Fontes et al. (21)	April 11, 2016	Case report	1	Brazil	Demyelinating	(+) in serum and urine (no details)	Cyto-albumin dissociation	Paraparesis; facial palsy (bilateral)

*AMAN, acute motor axonal neuropathy; bilat, bilateral; EDX, electrodiagnosis; GBS, Guillain–Barre syndrome; PRNT, plaque reduction neutralization test; RT-PCR, reverse transcription-polymerase chain reaction; (+), positive; (−), negative; unilat, unilateral*.

The authors of the case–control study reported that the diagnosis of GBS was based on the Brighton criteria (25), which requires the absence of identified alternative diagnoses for weakness. However, no differential diagnosis for a motor variant of GBS was presented, and there was no indication that mimics of GBS were identified or considered. This is a major omission because the absence of alternative diagnoses for weakness is the only diagnostic criteria in the Brighton criteria that is required for all four levels of GBS diagnostic certainty, ranging from level one (highest level of diagnostic certainty) to level four (reported as possible GBS due to insufficient data for further classification). Moreover, in the current clinical situation, non-poliovirus myelitis is one of the major differential diagnoses of motor variants of GBS (10, 11). The clinical features of the 42 GBS cases also merit comment. In this series, 48% (13/27) of patients with facial palsy had unilateral weakness on admission. It is unclear if asymmetrical weakness was also present in limb muscles because weakness was not expressed using Medical Research Council (MRC) scores or other GBS disability scales, and symmetrical weakness was not defined. Since GBS typically presents with symmetrical weakness, a patient series in which nearly half of facial palsy patients presented with unilateral weakness should raise concerns about alternative etiologies. Surprisingly, the majority of GBS patients (52%, 22/42) did not have areflexia or decreased reflexes at the nadir of the weakness. Given that loss of deep tendon reflexes is a classic feature of GBS that is required for three of four levels of GBS diagnostic certainty (Brighton levels 1–3), this finding alone casts doubts about the accuracy of the diagnosis. In the 42 GBS cases, 88% had a viral syndrome that preceded onset of neurological symptoms by a median of only 6 days. The disease was also characterized by rapid progression (median of 6 days from onset to nadir). However, as acknowledged by the authors (12), GBS typically occurs 2–8 weeks after an infection, and motor dysfunction progresses over up to a 4-week period. This is in agreement with the time lag between acute rash attributed to Zika virus and cases of demyelinating form of GBS in Brazil, which peaked 5–9 weeks after the rash (26). The cumulative clinical features of GBS cases from French Polynesia, including asymmetric weakness, preserved reflexes, early onset and rapid evolution of the paralysis, are not characteristic of GBS and warrant consideration of alternative etiologies. In fact, as a group, the clinical presentation is more compatible with non-poliovirus (Flavivirus) myelitis than the acute motor axonal neuropathy variant of GBS (6, 27). The fact that typical anti-ganglioside antibodies seen with acute motor axonal neuropathy variant of GBS were rarely present in cases from French Polynesia (12) also raises concerns about alternative pathogenesis of the paralysis.

The electrodiagnostic results demand even greater scrutiny, since these studies represent the most important laboratory test to confirm GBS variants and to differentiate between GBS and its mimics. Co-authors of the case–control study also published the first case report of GBS occurring immediately after a Zika virus infection (18), which overlapped the study period in the case–control study. However, the electrodiagnostic findings in the case report “confirmed a diffuse demyelinating disorder,” as seen in other case reports (Table 1), rather than the acute motor axonal neuropathy variant of GBS, as reported later (12). The needle electromyogram (EMG) in the first case report, performed only a few days after onset of neurological symptoms, reportedly showed “acute denervation, without axonal abnormalities” (18). However, this is an implausible finding because acute denervation implies axonal loss, does not occur in acute demyelinating disorders, and usually manifests after a latent period of 2–3 weeks following the onset of axonal degeneration (28, 29). The authors of the case–control study and the subsequent paper on the 42 GBS cases reported results of “sensitive” nerve action potentials, rather than “sensory” nerve action potentials. The lack of acceptable terminology raises doubts about the authors’ and the reviewers’ familiarity with basic principles of nerve conduction studies. EMG data were not reported in the case–control study, another noteworthy omission because the needle examination is needed to confirm degeneration of motor axons and to look for asymmetric denervation, a hallmark of Flavivirus myelitis (6). The authors also reported that nerve conduction studies showed the “same pattern in all tested nerves.” However, the various conduction abnormalities in all forms of GBS reflect the time at which studies are performed relative to disease onset and the temporal changes that occur in response to varying degrees of axonal degeneration and demyelination (27–29). Not surprisingly, serial conduction studies typically show large variability among different patients and even from one nerve to another in the same patient (28). Thus, it is difficult to conceive of consecutive GBS cases showing identical conduction abnormalities. In the case–control study, all 37 patients who underwent electrodiagnostic testing “during the first week of GBS onset” reportedly showed conduction abnormalities (12). However, in the acute motor axonal neuropathy variant of GBS, conduction studies are typically normal within the first week of symptoms but defined abnormalities evolve with serial studies that clarify the diagnosis (27, 28). In a series of 31 patients with the acute motor axonal neuropathy variant of GBS, distal compound muscle action potential (CMAP) amplitudes (i.e., amplitudes of motor nerve responses) performed within the first week of disease onset were normal in 69 and 60% of median and ulnar nerves, respectively, while distal motor latencies were normal in 72 and 100% of median and ulnar nerves (27). This is in stark contrast to the electrodiagnostic results presented in the case–control study, where all motor nerve responses showed marked reduction of the distal CMAP amplitude and prolonged distal latencies in the first week (12). The small number of motor nerve conduction studies performed (one motor nerve in lower limbs and two in upper limbs) precluded a search for asymmetric involvement and warrant an explanation how a motor variant of GBS was diagnosed in 18 out of 42 cases (43%) that presented with muscle weakness confined to lower limbs. Given that the electrodiagnostic evaluation is an extension of the neurological examination, it is insufficient to examine only one motor nerve in the paraparetic lower limbs and two motor nerves in the asymptomatic upper limbs. Indeed, a Position Statement by the American Association of Neuromuscular and Electrodiagnostic Medicine (29), endorsed by the American Academy of Neurology and the American Academy of Physical Medicine & Rehabilitation, reads: “In order to characterize the nature of a polyneuropathy (axonal or demyelinating, diffuse or multifocal) and in order to exclude polyradiculopathy, plexopathy, neuronopathy, or multiple mononeuropathies, it may be necessary to study four motor and four sensory nerves, consisting of two motor and two sensory nerve conduction studies in one leg, one motor and one sensory nerve conduction study in the opposite leg, and one motor and one sensory nerve conduction study in one arm. At least two limbs should be studied by a needle EMG examination.” The electrodiagnostic evaluations performed in French Polynesia clearly fall short of these guidelines. The interpretation of the available nerve conduction studies also conveys a limited understanding of the principles and practice of electrodiagnosis in neuromuscular diseases.

Data from the case–control study have been used to project the threat of Zika virus GBS in at-risk countries and to prepare for the anticipated GBS epidemic. A recent Viewpoint paper anticipating the challenges of Zika virus and the projected incidence of GBS in the United States (population 320 million) concluded that “as many as 30,000 cases of Zika virus-associated GBS might be expected,” assuming the risk of GBS of 0.24 per 1,000 Zika virus infections and a 66% attack rate in those at risk (30). If true, the costs of GBS immunotherapy in the United States alone could be 500 million to 1 billion dollars^4^. The authors recommended careful planning to ensure an adequate supply and distribution of intravenous immune globulin and to maximize plasmapheresis availability (30), the two evidence-based acute treatments for GBS. In contrast, recent concerns have arisen regarding the epidemiological data used in the case–control study to support a causal relationship between Zika virus infection and GBS. Correspondence directed at the epidemiology argued that the association between GBS and Zika virus infection probably resulted from confirmation bias (31), the unconscious attempt to justify a conclusion already drawn instead of impartially collecting and assessing evidence to come to a valid conclusion (32). An accompanying correspondence also concluded that the “measure of association between Zika virus and GBS might be spuriously overestimated, perhaps markedly” (33).

## Conclusion

On the basis of our review, we conclude that evidence for a causal relationship between Zika virus infection and GBS is insufficient because clinical, serological, and electrodiagnostic data failed to confirm an acute motor axonal neuropathy variant of GBS and exclude other possible causes of paralysis. The acute flaccid paralysis associated with Zika virus was attributed to a motor variant of GBS, without considering alternative explanations, and quickly endorsed by reviewers, editorial teams, and national and international public health organizations. It merits further discussion how a case–control study without a secure diagnosis survived the rigorous scientific review process to mobilize a worldwide effort to tackle an unproven threat. This is particularly pertinent because there are no other studies that provide validation that Zika virus is a cause of GBS. While there may still be an association between Zika virus and the demyelinating form of GBS, based on published case reports (Table 1), it remains unclear if these few case reports reflect a genuine increase in GBS precipitated by Zika virus, the result of enhanced reporting, or confirmation bias. The distinction between acute flaccid paralysis due to GBS versus other causes has global economic implications because each case misdiagnosed and treated as GBS may cost tens of thousands of additional dollars.

Heretofore, we suggest that intensified focus be redirected at differential diagnosis, proper use of diagnostic criteria, and electrodiagnostic evaluations that follow recommended guidelines. This will help to clarify the pathogenesis of Zika virus acute flaccid paralysis. In this effort, experts in neuromuscular disorders should play a more important role. Until pathogenesis is established, we propose that new cases of Zika-related weakness be termed “Zika virus acute flaccid paralysis,” rather than attributed to variants of GBS. We also call for a moratorium on recommendations for at-risk countries to prepare costly immunotherapies directed at GBS.

## Author Contributions

All authors listed, have made substantial, direct and intellectual contribution to the work, and approved it for publication.

## Conflict of Interest Statement

The authors declare that the research was conducted in the absence of any commercial or financial relationships that could be construed as a potential conflict of interest. The reviewer RB and handling Editor declared their shared affiliation, and the handling Editor states that the process nevertheless met the standards of a fair and objective review.

## Footnotes

^1^http://www.cdc.gov/

^2^http://ecdc.europa.eu/

^3^http://www.who.int/

^4^http://www.howmuchisit.org/how-much-does-ivig-cost/ (accessed August 1, 2016).
